# The molecular mechanism of Gorham syndrome: an update

**DOI:** 10.3389/fimmu.2023.1165091

**Published:** 2023-05-05

**Authors:** Juqin Xiang, Weiyang Zhong

**Affiliations:** ^1^ Chongqing Medical University, Chongqing, China; ^2^ Department of Orthopaedics, The First Affiliated Hospital of Chongqing Medical University, Chongqing, China

**Keywords:** Gorham syndrome, molecular mechanism, lymphoid proliferation, vascular proliferation, osteolysis treatment

## Abstract

Gorham syndrome, also known as “vanishing osteopathy” and “invasive hemangiomatosis,” is a rare clinical syndrome whose etiology is unknown and can invade the whole-body skeleton. At present, more than 300 cases have been reported at home and abroad, usually manifesting as spontaneous chronic osteolysis with no periosteal reaction at the lysis site and occult onset, often with fractures, scoliosis, chylothorax, etc. When waiting for medical treatment, the condition is serious, and the prognosis is poor. At present, there is no effective treatment. The main pathological manifestations of Gorham syndrome are the non-neoplastic abnormal proliferation of lymphatic vessels or blood vessels and osteolysis caused by osteoclast proliferation or increased activity. At present, there is no unified conclusion regarding Gorham syndrome’s pathogenesis. This paper starts with the two most studied osteolysis methods at present, osteoclast osteolysis and osteolysis caused by vascular and lymphatic proliferation and summarizes the corresponding most possible molecular mechanisms in recent years to provide more ideas for Gorham syndrome treatment.

## 1 Introduction

Gorham syndrome, that is, massive osteolysis, was first discovered by Jackson in 1838 and first reported by Gorham and Stout in 1955. It is characterized by progressive bone resorption, which occurs slowly or rapidly and is related to the proliferation of lymphatic vessels or blood vessels near osteolysis (1). It can occur from infancy to old age, and there are no gender or regional differences. Patients’ X-rays show transparent shadows, indicating bone destruction. Imaging findings usually include single or multiple osteolytic bone destruction, soap bubbles, or honeycomb changes with clear boundaries (2) and do not include osteomas, dead bone, or periosteal reactions in the osteolytic area. The maxillofacial region, upper limbs, clavicles, ribs, vertebrae, pelvis, skull, etc., are usually affected. Gorham syndrome affects multiple bones in most patients; only a few cases of a single affected bone have been reported. Vertebral body bone destruction caused by thoracic vertebrae invasion can weaken the lower limbs and cause chest and back pain (3). Osteolysis aggravation can lead to local deformities, which can invade the lower ribs, cause progressive kyphosis (2), thoracic duct injury, etc., and form many chylothoraces. This, combined with a unilateral or bilateral chylothorax, leads to a poor prognosis; most patients die of malnutrition and paraplegia (4–6). Most patients’ conditions are self-limited in the disease’s later stages. The osteolysis can be terminated by itself. At present, there is no specific index for Gorham syndrome diagnosis, which is an exclusive diagnosis that primarily depends on a combination of pathological and imaging examinations (7). In 1983, Heffez et al. proposed the following eight diagnostic criteria for osteolysis (8): a positive bone biopsy; little or no osteoblast response; no malnourished calcification; non-dilated non-ulcerative lesions; no visceral involvement; an osteolytic imaging model; local progressive bone resorption; no hereditary, metabolic, neoplastic, immunological, or infectious venereal etiology; and no cellular atypia.

## 2 Osteolysis mechanism dominated by osteoclasts

### 2.1 Receptor activator of nuclear factor-κB ligand

Osteocytes are the primary regulators of bone homeostasis between osteoclasts and osteoblasts. Osteocytes secrete cytokines (including sclerosin) and the Dickkopf-1(DKK-1)/Wnt pathway, RANKL, and osteoclastogenesis inhibitory factor (OPG) inhibitors, which respond to mechanical stimulation and initiate bone resorption and osteocyte death and can lead to osteoclast recruitment (9). Bone remodeling homeostasis is primarily maintained by RANKL and OPG (10). In healthy people, the RANKL/OPG ratio is stable. However, under lesion conditions, the RANKL/OPG ratio increases, which leads to an increase in bone resorption (11, 12). Osteoclasts are multinucleated cells that originate from the mononuclear macrophage lineage and cause bone resorption. Osteoclasts can be activated by different cytokines secreted by immune system cells. Monocyte/macrophage colony stimulating factor (M-CSF) and RANKL are responsible for the differentiation of progenitor cells into mature osteoclasts. M-CSF causes osteoclast formation, while RANKL contributes to differentiation and activation into mature osteoclasts (13).

**Figure 1:**
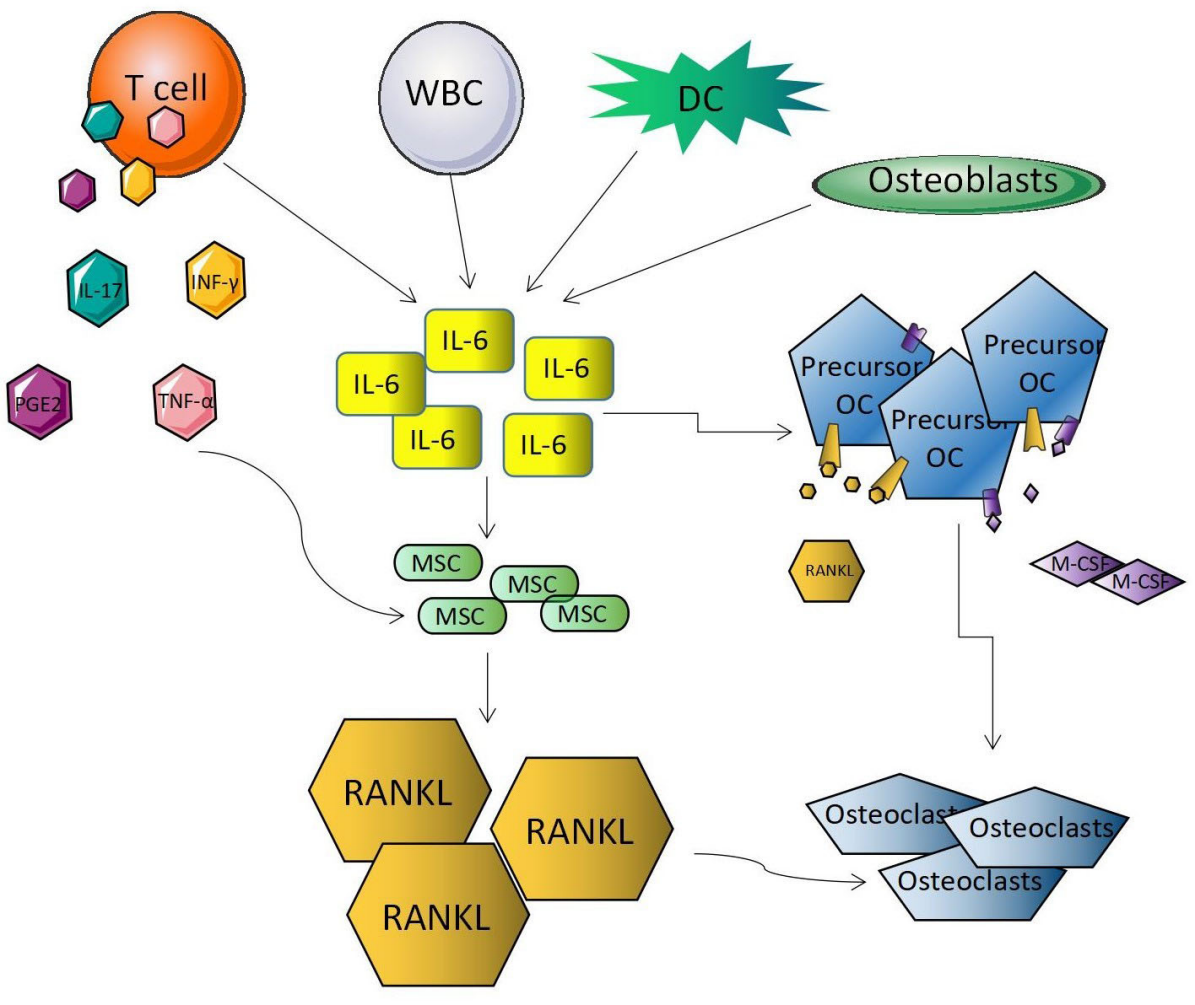
RANKL in osteolysis mechanism.

RANKL, a member of the tumor necrosis factor family, promotes osteoclast formation and differentiation through nuclear factor kappa-B (NF-κB) receptor activator binding, which is a key factor in bone resorption (14). T lymphocytes produce cytokines such as interferon-γ (IFN-γ), tumor necrosis factor-α (TNF-α), prostaglandin E2, and interleukin-17 (IL-17), which induce the activation of cytokine receptor activators of nuclear factor NF-κB ligand (RANKL). Studies (15) have shown that interleukin-6 (IL-6) increases progenitor cells’ sensitivity to humoral factors (RANKL and macrophage colony stimulating factor), which leads to the activation and differentiation of osteoclasts. In addition, T lymphocytes, white blood cells, osteoblasts, and dendritic cells produce IL-6, which activates mesenchymal stem cells in the bone marrow and stimulates RANKL production. RANKL binds to the RANK receptor expressed on osteoclast precursors leading to the differentiation and activation of osteoclasts (**Figure 1**). Studies have shown that lymphatic endothelial cells (LECs) can significantly increase the receptor activator of osteoclast formation and bone resorption mediated by RANKL. In one study, BM cells were cultured using MC-3T3E1 osteoblast precursor or BEC’s CM (LEC-conditioned medium) in the presence of RANKL. LECs CM induced more osteoclast formation than other CM cell types, suggesting that RANKL can increase the osteoclast formation potential induced by LECs (16).

### 2.2 Interleukin-6

IL-6 is a strong osteoclast-stimulating factor (17). Rossi et al. (18) found that as the number of osteoclasts increased, IL-6 in serum biochemical analysis increased compared with the normal level, and *in vitro* experiments showed that osteoclasts’ differentiation and activity increased. It can be speculated that IL-6 may participate in osteoclast osteolysis through an unknown mechanism. Other studies found that IL-6 serum levels significantly increased to seven times the normal upper limit in the early stages of treatment, whereas after treatment, IL levels decreased to 1/4 of what they were before treatment (19). That IL-6 was significantly higher than the normal value and then decreased during treatment suggests that IL-6 is involved in osteolysis and that the course of osteolysis may be blocked by inhibiting IL-6 (20). Some scholars believe that IL-6 increases the sensitivity of osteoclast precursors to RANKL and M-CSF through the RANKL pathway (21), accelerates the differentiation and maturation of osteoclast precursors induced by M-CSF and RANKL, and leads to bone resorption (22, 23). In most studies, IL-6 was higher than the normal value (8, 24, 25), but IL-6 was not elevated in all cases (16); therefore, IL-6 cannot be used as an indicator to diagnose osteolytic disease.

**Figure 2:**
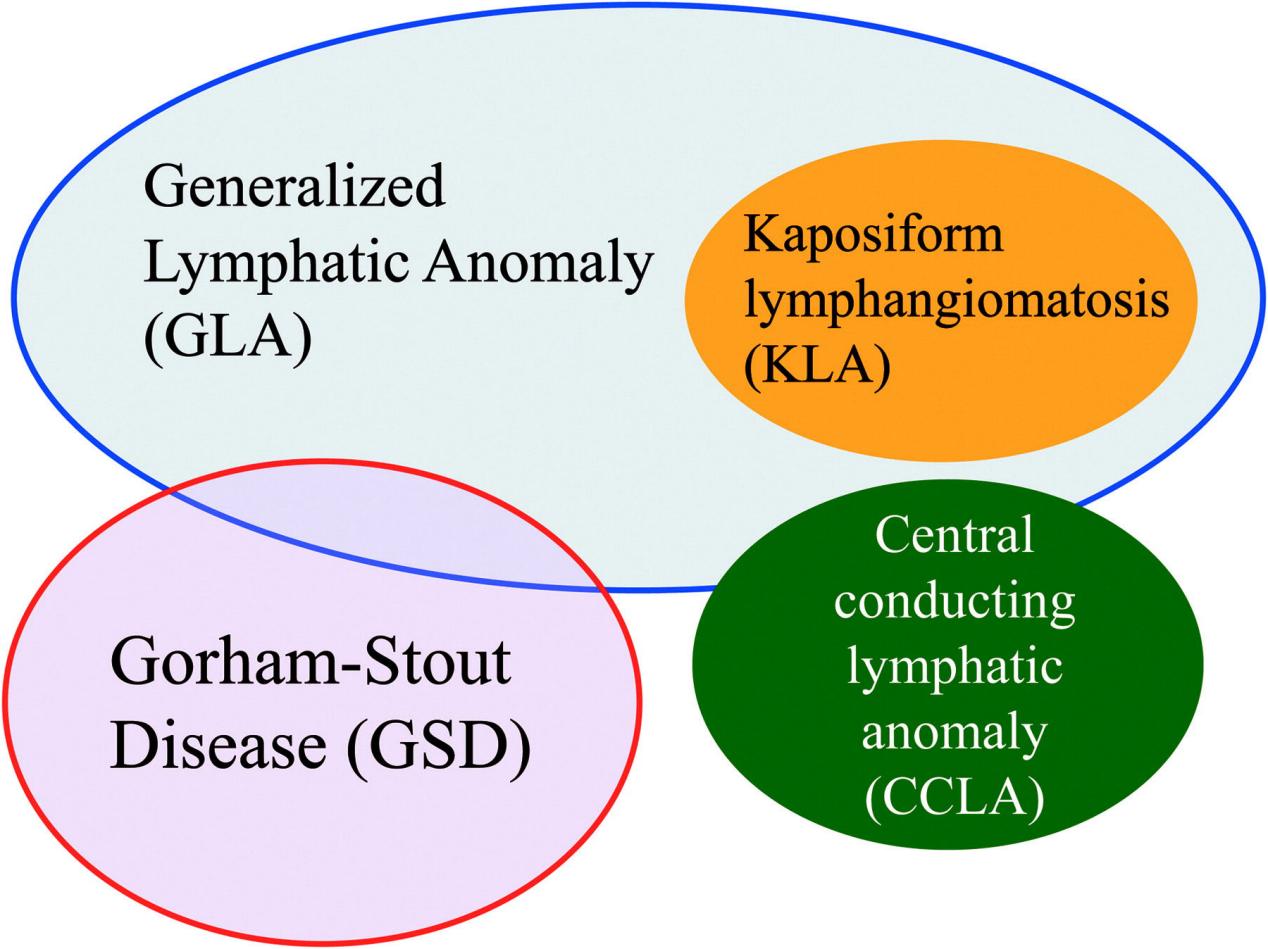
Overlapping characteristics of complex lymphoid abnormalities.

It has been controversial whether osteoclasts, as osteolytic cells, are involved in the bone destruction mechanism. Osteoclasts, the only cells with an osteolytic effect, occupied a dominant position from the very beginning. Later, Gorham and Stout thought that osteoclasts were unnecessary. Foult et al. found that osteolysis was secondary to hemangiomatosis. However, Spieth et al. reported an obvious relationship between osteoclasts and this rare syndrome, and Möller et al. found many multinucleated osteoclasts with hyperactive reabsorption function in patients (26). These studies have not clarified whether osteoclasts are involved in Gorham syndrome osteolysis. Some studies suggest that there is no local increase in the number of osteoclasts. For example, in a case analysis (26), osteoclasts were found in only five of 67 patients, suggesting that osteoclasts may not be a necessary condition for osteolysis. As for the mechanism of osteoclast involvement, Ray et al. (27) noted that osteoclast precursors may be more sensitive to humoral factors, which promote osteoclast formation and bone resorption at the bone microenvironment level. Rossi et al. (28), proposed that Treg cells in Gorham syndrome patients could still inhibit osteoclast activity and osteolysis by enhancing the effect of Treg cells. In recent years, studies (5, 18) have shown that Gorham syndrome’s pathogenesis results primarily from osteolysis caused by non-neoplastic proliferation of blood vessels and lymphatic vessels. With deepening research, the main point of view is that Gorham syndrome is related to the excessive growth of lymphatic vessels in proliferative vessels. Lymphatic endothelial cells may secrete factors that affect the activity of osteoclasts and/or osteoblasts. The uncontrolled growth of fluid-filled lymphatic vessels may cause osteolysis by pressing bone, and mechanical stimulation may play a role in this. The effect of osteoclasts is not obvious. In 2014, the International Society for the Study of Vascular Anomalies (ISSVA) updated Gorham syndrome’s classification, which was approved at its twentieth seminar. Gorham syndrome is classified as a complex lymphatic abnormality (CLA) because of its many overlapping clinical features with other diseases in the CLA family (**Figure 2**) (29).

## 3 Nomenclature lymphatic and angiomatous hyperplasia

### 3.1 D2-40

D2-40 can be expressed on the surfaces of lymphatic endothelial cells and mesothelial cells (30). D2-40 can also be used to identify whether the fissures around a tumor are lymphatic vessels. At present, D2-40 monoclonal antibody immunohistochemical staining is considered a highly sensitive and specific marker of the lymphatic endothelium. Choi et al.’s (31) study included a patient with a history of black stool, abdominal pain, and back pain. In a duodenal biopsy, mucosal and submucosal lymphatic vessels were significantly dilated, and D2-40 staining was positive, indicating lymphoid or lymphatic venous malformations. During the examination, the patient had dyspnea. An X-ray examination revealed a pleural effusion. A pleural biopsy showed thin-walled blood vessels of various shapes and sizes, lined with endothelial cells, which were positive using D2-40 immunohistochemical staining. After chylothorax drainage and corresponding symptomatic support treatment, the patient’s back pain and abdominal pain weakened, and his condition tended to be stable. In another study (32), pathological biopsies were performed on the pleural and abdominal puncture fluid of a patient with chylothorax and chyloma. Results showed that the lymphatic vessels were dilated in the proliferative fibrous connective tissue, and there was lymphocyte infiltration in the trabecula. The immunohistochemical staining of lymphatic endothelial cells in the dilated lymphatic vessels was strongly positive for D2-40. Other studies (33) noted that at a lesion’s early stage, tissue biopsies of bone defect areas often showed nonspecific vascular hyperplasia, surrounded by hyperplastic fibrous connective tissue. Immunohistochemical staining showed positive expression of CD31 (34), CD34 (35), and D2-40 in hyperplastic vessels’ endothelial cells (36, 37). Although such tissue is benign, it tends to invade and destroy adjacent tissue. Recently, many studies (38–40) have found that D2-40 or LYVE-1 staining of thin-walled vascular endothelial cells in cancellous bone, cortical bone, and soft tissue in the focus of bone autolysis is positive, whereas that of normal bone tissue is negative. Together, the above studies strongly suggest the presence of many proliferated lymphatic vessels in the diseased area, which may be related to osteolysis occurrence.

### 3.2 VEGF (vascular endothelial growth factor)

Many studies have noted that higher than normal levels of vascular endothelial growth factor (VEGF) were detected in most Gorham syndrome patients’ lesion areas (18, 19, 25). VEGF was first discovered by Senger et al., who called it vascular permeability factor (VPF); it is a physiological and pathological angiogenic factor (41). It can improve hypoxia during tumor growth and promote angiogenesis and tumor progression (42). It is important in new blood vessel formation and can promote the differentiation of mesenchymal stem cells into vascular and lymphatic endothelial cells. VEGF signal transduction plays an important role in angiogenesis and lymphangiogenesis. VEGFR-3, the VEGF-C/D receptor, is a key lymphangiogenesis regulator. A high local concentration of VEGF-C or other lymphangiogenic factors (such as VEGF-A, VEGF-D, and angiopoietin) can induce angiogenesis and lymphangiogenesis in Gorham syndrome patients’ bone marrow (43). Some articles (17) indicate that sirolimus can inhibit the growth of abnormal lymphatic vessels and reduce pathological changes in Gorham syndrome patients; it has no adverse effect on normal lymphatic vessels. Notably, VEGFR-3 and Prox1 decreased in treated lymphatic endothelial cells (44), further indicating that VEGF is involved in the osteolysis process. Views differ regarding VEGF subtypes’ effects on Gorham syndrome. Ozeki et al. reported that VEGF-A, -C, and -D plasma levels in patients with lymphoid malformation were significantly higher than those in a control group; it was also noted that the VEGF-A, VEGF-C, and IL-6 serum levels in Gorham syndrome patients were all increased. It has been demonstrated in a paper (45) that RANKL induces osteoclasts to express the lymphatic growth factor, VEGF-C, and that VEGF-C, by binding to its receptor, VEGFR3, on osteoclasts, directly increases osteoclastic bone resorption without affecting osteoclast formation or survival. Baud et al. (46) found that inflammation-induced lymphangiogenesis could effectively be blocked by the systemic injection of a VEGF-A neutralizing antibody; however, this phenomenon was not observed using VGEF-C. In view of this difference, the authors explained that lymphoid malformation and Gorham syndrome are not the same clinical entity and proposed that Gorham syndrome depends on VEGF-A, not VEGF-C or FLT-1. In addition, this case shows that propranolol can be administered safely and has a significant therapeutic effect on Gorham syndrome by reducing circulating VEGF-A rather than by changing VEGF-C or Fms-like tyrosine kinase-1 (FLT-1) levels. In another article, the authors noted that patients had elevated VEGF levels but did not show significant osteolysis or chylothorax, which subsequently decreased after treatment with interferon-α2b. Other studies have noted that VEGF and VEGF-C levels in one patient decreased from slightly higher to normal during treatment, whereas another patient’s levels were not significantly abnormal (47). VEGF changes provide a promising treatment for Gorham syndrome patients. Most studies mention an increasing VEGF complex trend but do not indicate which VEGF subclasses changed; exploring changes in different VEGF subtypes requires additional supporting data.

### 3.3 Endoglin (CD105, cluster of differentiation 105)

Endothelial glycoprotein (CD105) is a transforming growth factor-β (TGF-β) family receptor that can be used as a coreceptor of TGF-β protein superfamily members and plays a key role in fibrosis and angiogenesis (42). Detecting CD105 in the diseased tissues of Gorham syndrome patients helps identify vascular proliferation. Franchi et al. (48) found that the expression level of the vascular endothelial marker CD105 in the diseased tissues of Gorham syndrome patients was significantly higher than that in hemangioma patients and normal infants and adults. This indicates that CD105 participates in osteolysis through some mechanism in Gorham syndrome patients but does not specify which mechanism is involved (23). Another study (23) noted that Gorham syndrome patients’ endothelial cells highly express CD105, which activates bone macrophages by producing IL-1 and then promotes angiogenesis and lymphangiomatosis *via* VEGF and TGF-β produced by bone macrophages. Together, these studies suggest that CD105 can be highly expressed in Gorham syndrome patients. Given the current lack of research and the preliminary exploration of CD105’s participation mechanism, there is no unified view at present.

### 3.4 Inositol phosphate 3-kinase pathway

**Figure 3:**
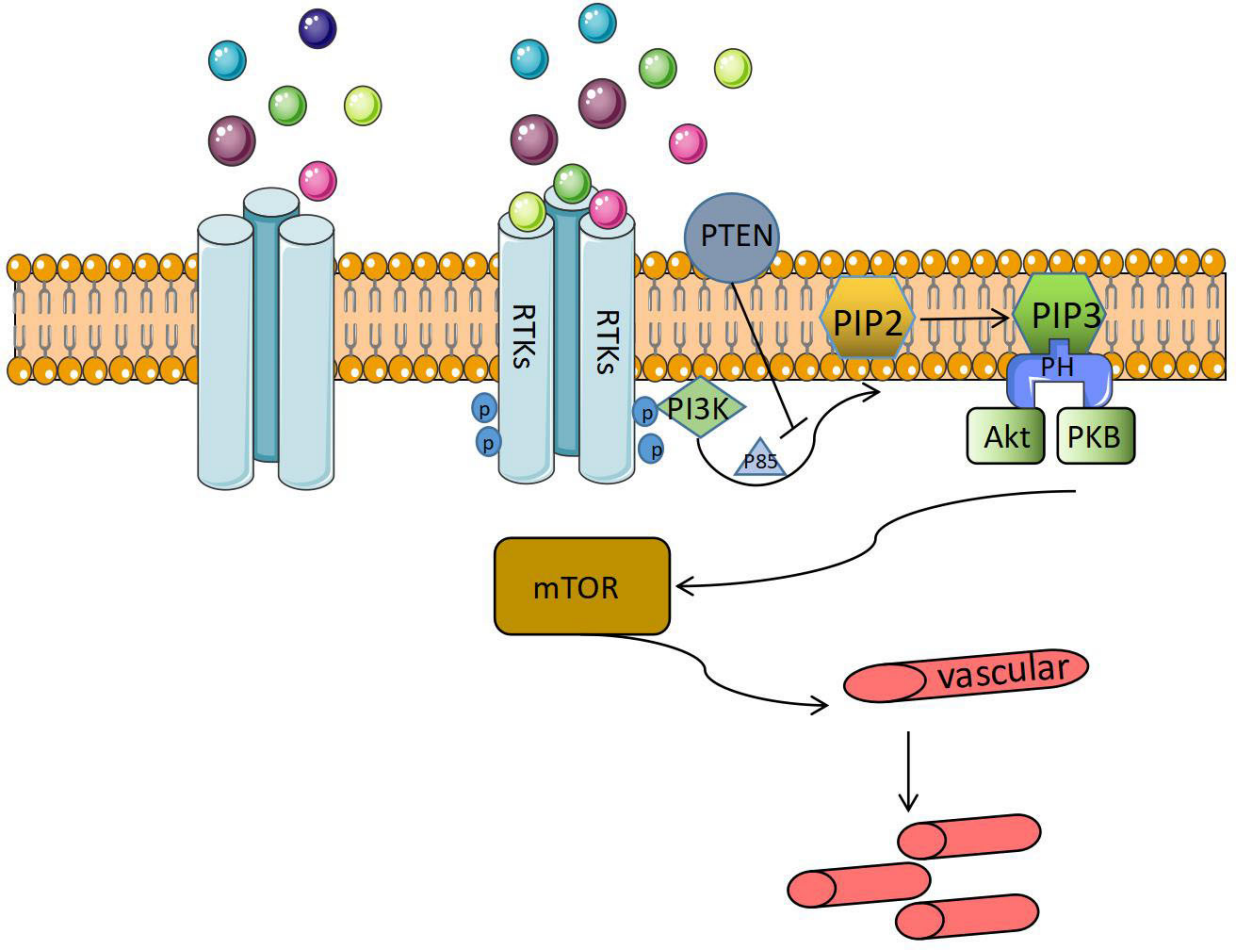
Anti-apoptotic pathway.

In recent years, it has been found that gene mutations of the phosphoinositide 3-kinase (PI3K) pathway and somatic mutations of the NRAS gene in the lymphatic endothelium may cause Gorham syndrome’s extensive lymphadenomegaly. Ozeki et al. (22) considered that Gorham syndrome pathogenesis might be related to the RAS/PI3K/mTOR component gene signaling pathway’s somatic mutation. The PI3K/Akt pathway plays a role in many cell processes, from cell cycle regulation to proliferation and migration. It is often called the anti-apoptotic pathway (**Figure 3**).

**Figure 4:**
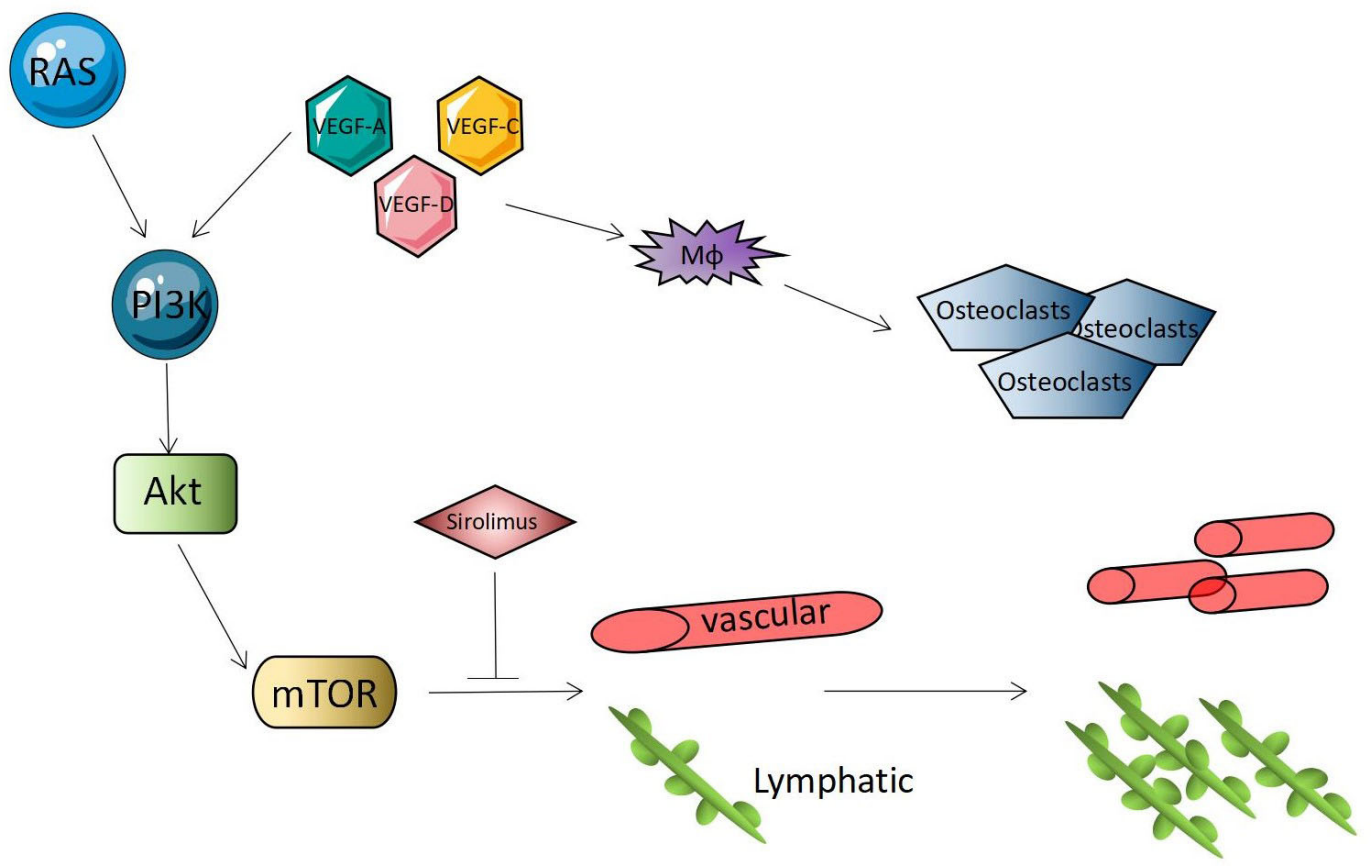
RAS-PI3K-Akt-mTOR signal pathway.

When the ligand binds to the receptor tyrosine kinase, it is triggered; the receptor tyrosine kinase is phosphorylated and attracts PI3K to the plasma membrane. PI3K directly interacts with receptors or connectors in its SH2 domain, such as phosphate tyrosine residues on P85, to open catalytic subunits. Phosphatidylinositol-4-diphosphate (PIP2) is converted to phosphatidylinositol-3-pyrrosine-4-trisphosphate (PIP3), which recruits pleckstrin homology (PH) domain-containing proteins, protein kinase B (PKB), and AKT (49) to the membrane to form a complex that can directly regulate the cell cycle and continue to activate the signal transduction target of rapamycin (mTOR) in mammals. PI3K/Akt/mTOR signal transduction is negatively regulated by the phosphatase PTEN. PTEN is a dual-specific phosphatase that can inhibit PI3K’ lipid activity. An absence of PTEN leads to a lack of dephosphorylation of the lipid substrate PIP3, which leads to the permanent stimulation of the PI3K/Akt/mTOR pathway, which leads to the proliferation of blood vessels (41, 50, 51). Some studies (30, 38, 52–54) proposed that mTOR is a serine threonine kinase regulated by phosphoinositide 3 kinase (PI3K) and protein kinase B (Akt). The PI3K-Akt-mTOR signaling pathway is closely related to cell growth and proliferation, increases the expression of VEGF, and regulates angiogenesis and lymphangiogenesis. The mTOR inhibitors can block downstream protein synthesis and have anti-tumor and anti-angiogenic effects (**Figure 4**). Gorham syndrome patients recovered completely using sirolimus (an mTOR inhibitor) to inhibit the RAS/PI3K/mTOR signaling pathway. Some studies (55) have shown that sirolimus can inhibit the growth of lymphatic malformations without affecting healthy lymphatic vessels. It has been confirmed that PI3K-Akt-mTOR plays a role in Gorham syndrome pathogenesis, which provides a theoretical basis for using sirolimus in the clinical treatment of Gorham syndrome.

### 3.5 Platelet-derived growth factor receptor signal pathway

Platelet-derived growth factor (PDGF) was initially found to be a serum growth factor for fibroblasts, smooth muscle cells, and glial cells. It binds to its receptors (PDGFR-α and PDGFR-β) through disulfide bonds to form a dimer to play the role of a tyrosine kinase. PDGF can promote angiogenesis and stimulate the growth and development of adjacent connective tissue cells, but only low PDGF expression and its receptors can be detected in normal blood vessels (56). One study found that platelet-derived growth factor receptor (PDGFR-β) was present in endothelial cells and most capillary structures directly adjacent to lesions in Gorham syndrome patients but not in non-Gorham lymphangiomatosis or normal skin or pleura used as controls (19). The study (57) showed that the PDGFR content in Gorham syndrome patients was significantly higher than that in normal subjects. It was suggested that the PDGFR signaling pathway’s mechanism may play a role in capillary proliferation, and the specific mechanisms may include activating phospholipase C-γ (PLC-Cγ), mitogen-activated protein kinase (MAPK), Src family tyrosine kinase (SFK), PI3K, etc., promoting cell proliferation and differentiation, and inhibiting cell apoptosis.

### 3.6 Other mechanisms

According to the pathological manifestations of osteolysis, the main Gorham syndrome mechanism at present is the abnormal proliferation of vessels. In previous studies, an increase in the proliferation and activity of osteoclasts were considered the dominant mechanisms. With the increase in case studies, Gorham syndrome’s cause is increasingly considered to be vascular proliferation, whereas the latest study indicates that it is primarily caused by lymphatic proliferation (58); however, there is still no definite data regarding the pathogenesis of osteolysis. In addition to the molecular mechanisms already discussed, there are less studied mechanisms; Keyser et al. (16) noted that macrophages inhibit osteoblast function by producing TNF-α, and that growth factors VEGF-A and VEGF-C and platelet-derived growth factor play a role in lymphatic invasion. These growth factors signal in the same way, which eventually works through the rapamycin target (mTOR). An important kinase in cell cycle progression, mTOR is also a key immune response regulator. TNF, TGR, LYVE-1, and other substances can abnormally increase in patients with osteolysis by affecting the lymphatic system’s proliferation and function. An increase in local blood flow in the lysis area, a change in pH, or an increase in mechanical pressure can lead to endothelial cell proliferation and promote bone loss. In contrast, Heyden et al. believe that a decrease in blood flow in the osteolysis area may lead to local hypoxia, reduce tissue pH, increase the activity of various hydrolases, and promote osteolysis (13). The exact nature of the pathological process of the disease is not clear. Gorham and Stout pointed out that hyperemia, local changes in pH, and mechanical forces caused bone resorption and excluded any role played by osteoclasts (59). For osteolysis caused by vascular proliferation, Ahmetgjekaj, Ilir et al. (22) speculated that wide capillaries are characteristic of lesions, with abnormally slow flow and changes in adjacent soft tissues, which may be due to lymphangiogenesis extending to the surrounding tissue. It was speculated that these structural features promote local hypoxia, lead to excessive production of hydrolase, and may lead to bone resorption.

## 4 Future prospects

At present, whether in the early or progressive stage of osteolysis, there is no effective treatment (60), but only symptomatic treatment and adjuvant therapy for Gorham syndrome. Osteolysis treatment primarily consists of inhibition of osteolysis, inhibition of lymphatic system proliferation, promotion of bone repair, and prevention and treatment of complications. There are three main options for Gorham syndrome treatment (39, 47), including surgical stabilization, radiotherapy, and drug therapy. Radiation is usually used to alleviate Gorham syndrome. There is currently no Gorham syndrome medical treatment approved by the Food and Drug Administration. Medical options that are not approved include bisphosphonates (61, 62) and/or sirolimus (63), propranolol, and interferon alpha (64). To treat chylothorax, some people have tried chemotherapeutic drugs and low-molecular-weight heparin. The disease was relieved by the combined use of teriparatide, sirolimus, and bisphosphonate. The disease rarity and treatment data limitations regarding the case series limit care standards to supportive surgical intervention and drug therapy. Drug therapy primarily uses lymphatic endothelial cell marker inhibitors (65). An operation has been performed to stabilize a GSS (general spine system) fracture (66, 67). Other treatments include radiotherapy and drug therapy (anti-osteoclast, IFN-α-2b, zoledronic acid, etc.) (68). During fusion, prostheses should be used instead of bone grafts to avoid repeated bone resorption. Some scholars have proposed that miRNA, which can regulate cell activity and vascular proliferation and has been found in osteoclasts and osteoblasts in Gorham syndrome patients, provides a new treatment option (69). At present, no specific drug has been identified for osteolysis treatment. If major hospitals share their patients’ clinical manifestations and primary disease treatment complications to clarify its pathogenesis as soon as possible, this will be conducive to research regarding therapeutic drug application.

## 5 Conclusion

In conclusion, Gorham syndrome is a rare disease with infrequent cases and a very low cure rate, all of which make it difficult to study its pathogenesis. Although many studies have explored Gorham syndrome from different perspectives, there is no unified conclusion to clarify the pathogenesis of osteolysis. With technological development and an increase in research data, some highly recognized viewpoints have been gradually formed, including lymphatic proliferation and vascular proliferation. However, whether osteoclasts are involved in osteolysis has become a polarized debate. Some researchers believe that osteoclasts, as the only osteolysis cells, must play a role in it. Other researchers believe that no changes in osteoclasts were found in Gorham syndrome-associated lesions and that osteoclasts are not involved in osteolysis’ pathological changes. Osteoclast-stimulating factors, including RANKL, IL-6, MIP-1-α, TNF-α, IL-3, and IL-11, can stimulate osteoclast differentiation and maturation to increase bone resorption through elevated levels. However, studies regarding RANKL’s involvement in osteolysis focus mainly on multiple myeloma, osteoporosis, breast cancer, etc. No recent studies have identified a necessary relationship between RANKL and Gorham syndrome. As an osteoclast-stimulating factor, IL-6 shows different pathological changes in different studies. Additional studies are required to determine whether it is involved in osteolysis as well as its mechanism. There are three views regarding Gorham syndrome pathogenesis: lymphatic proliferation, vascular proliferation, and a combination of the two; there is no unified conclusion.

Vascular proliferation is currently a frontier research direction. CD105, VEGF, D2-40, the PI3K pathway, and the PDGFR pathway can all cause vascular or lymphatic proliferation. Some theories argue that a significant amount of vascular proliferation and compression of bone tissue results in osteolysis, whereas another theory is that microenvironmental changes caused by vascular proliferation induce bone resorption; there is no consistent point of view. For this pathogenesis, current treatment methods include interferon therapy, chemotherapy, immunosuppression, and immunotherapy, all of which have achieved a degree of curative effect. Starting with the mechanisms so far identified, this paper summarizes the molecular mechanisms of osteolysis, which have been widely studied, to provide a reference for the study of osteolysis’ pathogenesis.

## Author contributions

WZ conceived and designed the study. JX collected and analyzed the data. JX wrote the manuscript. WZ revised the draft. All authors contributed to the article and approved the submitted version.
